# Frequency of Sexual Problems and Related Psychosocial Characteristics in Cancer Patients—Findings From an Epidemiological Multicenter Study in Germany

**DOI:** 10.3389/fpsyg.2021.679870

**Published:** 2021-07-22

**Authors:** Svenja Heyne, Peter Esser, Kristina Geue, Michael Friedrich, Anja Mehnert-Theuerkauf

**Affiliations:** Department of Medical Psychology and Medical Sociology, University Medical Center Leipzig, Leipzig, Germany

**Keywords:** anxiety, cancer, distress, epidemiology, oncology, quality of life, screening, sexual problems

## Abstract

**Background:**

Multimodal cancer treatments are often associated with sexual problems. Identifying patients with sexual problems could help further elucidate serious issues with their sexuality and thus promote or maintain patients’ sexual health. We aimed to assess the occurrence of sexual problems in patients across different tumor locations and to explore associated sociodemographic, medical and psychosocial factors.

**Methods:**

We included 3,677 cancer patients (mean age 58 years, age range 18–75 years, 51.4% women) from a large epidemiological multicenter study in Germany on average 13.5 months after cancer diagnosis. The occurrence and frequency of sexual problems were assessed via a binary item on the problem checklist of the Distress Thermometer (DT). Controlled associations of these problems with sociodemographic, medical and psychosocial factors including distress (DT), anxiety (GAD-7), depression (PHQ-9), quality of life (EORTC-QLQ-C30), and social support (SSUK-8) are analyzed using logistic regression analysis.

**Results:**

We found that 31.8% of patients reported sexual problems, with a significant higher proportion in men (40.5%) compared to women (23.7%), OR 2.35, 95% CI [1.80–3.07] and a higher proportion in patients with a partner (35.6%) compared to those without a partner (3.5%), OR 2.83, 95% CI [2.17–3.70]. Tumor location was associated with occurrence of sexual problems: patients with cancer, affecting the male genital organs had the highest chance for sexual problems, OR 2.65, 95% CI [1.18–3.95]. There was no significant difference in the occurrence of sexual problems between age groups OR 0.99, 95% CI [2.13–3.53] and type of therapy (e.g., operation OR 0.91, 95% CI [0.72–1.15]). Sexual problems were further associated with elevated levels of anxiety, OR 1.05, 95% CI [1.02–1.10], less social support, OR 0.93, 95% CI [0.90–0.97] and lower quality of life in terms of impaired functioning (e.g., social function, OR 0.99, 95% CI [0.99–1.00]).

**Conclusions:**

Sexual problems are commonly reported by patients. Male patients and those living with a partner are more likely to report sexual problems. Sexual problems are associated with different aspects of well-being. The findings imply the practical relevance to screen for sexual problems among patients and identified groups that should be particularly monitored.

## Introduction

Detrimental effects on sexuality through cancer and its treatment are common (Zebrack et al., 2010; Bober et al., 2013). Long-term and late effects on sexual functions may occur in both men and women. For example, decreased sexual sensation and sexual responsiveness affects over 60% of women diagnosed with cancer (Valpey et al., 2019), while erectile dysfunction affect up to 75% of men with cancer (Shpot et al., 2018). To date, however, previous findings are mixed. Regarding the prevalence of sexual problems in men and women, several studies found that gender has an influence on sexual health (Koyama, 2016; Reese et al., 2018) whereas other studies showed no gender differences (Krok et al., 2013).

Existing research has mainly focused on women who have breast or gynecological cancer (Chen et al., 2018; Ljungman et al., 2018; Condorelli et al., 2019) and men who have prostate cancer (Cakar et al., 2013; Fode et al., 2017; Clavell-Hernández et al., 2018) as well on mixed populations such as colon cancer patients (Canty et al., 2019; Stulz et al., 2020). Some cancer types, including gynecological or prostate cancer directly affect the sexual organs. However, sexual functioning can also be affected indirectly by various cancer treatments (Arden-Close et al., 2011). Surgical treatments like mastectomy or orchiectomy or the placement of a temporary or permanent stoma can adversely affect a healthy body image (Esser et al., 2018) and therefore lead to changes in sexual self-esteem (Sun et al., 2018). This might have a long-lasting effect on cancer patients sexuality in consequence of anatomical defects incurred, whereas sexual dysfunctions caused by chemotherapy and radiotherapy mostly disappear after terminating treatment (Cakar et al., 2013). In the case of hormonal treatment, a significant number of cancer patients become menopausal during active cancer treatment, and postmenopausal symptoms often remain stable after active cancer therapy (Mourits et al., 2002). Also hormonal treatment (e.g., androgen deprivation therapy) can lead to erectile dysfunction in men with long-lasting effects even after termination of the therapy (Schover, 2015).

To explore the relevance of sexual problems within cancer patients it is important to identify its association with psychosocial problems and quality of life. Previous studies show symptoms such as depression, fatigue, and fear of recurrence have an adverse impact on physical and psychosocial functioning and overall quality of life (Yi and Syrjala, 2017), as well as on sexual quality of life (Archangelo et al., 2019) and quality of the relationship between both partners (Carter et al., 2018). It is unclear, whether anxiety, depression, and distress as independent factors influence sexuality, sexual problems and the overall sexual health. Previous studies found associations between medical factors and sexual dysfunction in colorectal cancer patients (Milbury et al., 2013) and in breast cancer patients (Gass et al., 2017) and demographic and sexual function in rectal cancer patients (Au et al., 2012). Although those studies could show a relationship between medical and sociodemographic factors that may be associated with sexual problems, data is limited on this across different tumor locations. However, data on this may help to identify those, which may particularly be vulnerable for sexual problems and to provide psychosocial care as early and as tailored to their specific needs as possible.

To address some of the limitations outlined above, we aimed to assess the occurrence of sexual problems in patients among a large representative sample across different tumor locations. We further aimed to analyze the association between sexual problems and medical and sociodemographic factors as well as with dimensions of psychosocial distress and quality of life in order to gain new knowledge that might help tailoring future interventions on sexual health in cancer patients.

## Materials and Methods

### Sample and Procedure

We used the sample of a large epidemiological cross-sectional multicenter study across all tumor locations on the prevalence of mental disorders and psychosocial distress in cancer patients, the methods of which are described in detail elsewhere (Mehnert et al., 2012, 2014). In the original study, we enrolled 4,020 cancer patients at 30 hospitals, cancer care clinics and rehabilitation centers in Germany. Here, for the secondary analyses, we selected cancer patients with available data on the one-single item “sexual problems” from the problem list of the Distress Thermometer (Mehnert et al., 2006).

Patients were eligible for study participation if they were diagnosed with any malignant tumor according to the medical record and/or their treating physicians’ evaluation, aged between 18 and 75 years, fluent in the German language and void of severe physical, cognitive and/or verbal impairments that interfered with a patient’s ability to give informed consent. Patients were given the questionnaires in the treatment center, including a pre-stamped envelope to be returned within 2 weeks.

The study complied with the Declaration of Helsinki and was approved by the Ethics Committees of all participating centers (file numbers: Freiburg 244/07; Hamburg: 2768; Heidelberg: S-228/2007-50 155 039; Leipzig: 200-2007; Schleswig-Holstein: 61/09 and Würzburg: 107/07). All participants provided written informed consent and data were processed according to German data protection laws (§§ 27-30a BDSG).

### Measures

*Sexual problems* were assessed using the respective binary item (yes/no) on the problem checklist of the validated German version of the National Comprehensive Cancer Network’s Distress Thermometer (NCCN DT, Mehnert et al., 2006). This item is one of the 36 potential causes of distress that are grouped in five subscales, i.e., (1) practical problems, (2) family problems, (3) emotional problems, (4) spiritual/religious concerns, (5) physical problems and an open answer option for possible other problems.

*General distress* was measured via the single item of the NCCN DT, a brief screening tool for cancer patients to assess distress on a visual analog scale ranging from 0 (no distress) to 10 (extreme distress). A score of 5 is internationally recommended as an indicator that a patient is distressed and needs support.

*Depressive symptomatology* was assessed using the validated German version of the Patient Health Questionnaire (PHQ; Löwe et al., 2002, 2004). 9 Items assessing the frequency of depressive symptoms and can be scored on a four-point Likert scale from 0 (“not at all”) to 3 (“nearly every day”). Higher scores indicate higher severity of depression (Löwe et al., 2004).

*Anxious symptomatology* was assessed using the validated German version of the Generalized Anxiety Disorder Scale (GAD-7; Spitzer et al., 2006; Löwe et al., 2008). Seven Items assess the frequency of symptoms of Generalized Anxiety disorder scoring on a four-point Likert scale from 0 (“not at all”) to 3 (“nearly every day”). Higher scores indicate higher severity of anxiety (Löwe et al., 2008).

*Quality of life* was assessed with the EORTC-QLQ-C30—the German version of the European Organization for Research and Treatment of Cancer Quality of Life Questionnaire (Jocham et al., 2009). We used the five functioning scales, i.e., (1) physical, (2) roles, (3) cognitive, (4) emotional, and (5) social functioning. Each item is rated on a four-point scale ranging from 1 (“not at all”) to 4 (“very much”). Additionally, a global health sub-scale scoring on a 7-point linear analog scale was used. Higher scores in the five functional scales and global health status scale represent better functioning (Aaronson et al., 1993).

We measured patients’ *need for social support* with the short version of the SSUK-8—Illness-specific Social Support Scale (Ullrich and Mehnert, 2010). Eight items are scored on a five-point scale from 0 (“never”) to 5 (“always”) and form the two subscales “positive support” and “detrimental interaction.” Higher values represent higher levels of positive support and detrimental interactions, respectively (Ullrich and Mehnert, 2010).

We obtained *clinical characteristics* from patients’ medical charts and sociodemographic characteristics were assessed by patients’ self-reports.

### Statistical Analysis

We applied descriptive analyses (percentages) to provide the occurrence of sexual problems with respect of sociodemographic and medical characteristics—including sex, age (grouped), marital status, living with a partner, work situation and tumor location, cancer care setting, classification of tumor development according to UICC (Union for International Cancer Control) stage. We applied multivariate analysis of variances (MANOVA) to test associations of sexual problems with levels of depression, anxiety, social support and quality of life.

To identify robust and independent associated factors, we subsequently applied a final analysis with a logistic regression model in which we entered the following six blocks of predictors into the equation: (1) sociodemographic factors, (2) psychosocial factors, (3) social support, (4) quality of life, (5) cancer location, and (6) type of therapy. Predictors were checked for multicollinearity with correlation analysis. Correlations ranged from |*r*| = 0.00 to |*r*| = 0.65 (EORTC scales—roles functioning and physical functioning). No correlations were above *r* = 0.7 (considered as cut-off for multicollinearity). In all analysis two-sided *p* < 0.05 were considered significant. Data analyses were performed with IBM SPSS Statistics 26 (IBM Corp., 2019).

## Results

### Participants

In total, 4,020 participated (response rate: 69.5%), on average 13.5 months post current cancer diagnosis. Participants were recruited in acute care hospitals (43%), outpatient units (33%) and inpatient rehabilitation centers (24%). We included all patients who completed the sexual problems item on the DT (*n* = 3,677 patients). Patients who did not complete the item “sexual problems” were female, older and living with a partner. For details of the sample (see Table 1).

**TABLE 1:** Sociodemographic and medical factors of cancer patients stating sexual problems compared with total sample.

	Total sample	Sexual problems “Yes”
	*n* (%)	*n* (%)
**Total sample**	3,677 (100.0)	1,170 (31.8)
**Sociodemographic factors**		
*Sex*		
Male	1,787	723 (40.5)
Female	1,890	447 (23.7)
***Age in years^*a*^***		
18–35	150	37 (24.7)
36–45	376	110 (29.3)
46–55	839	283 (33.7)
56–65	1140	380 (33.3)
66–75	1119	340 (30.4)
***Marital status^*a*^***		
Single	442	100 (22.6)
Married	2,554	904 (35.4)
Divorced	382	100 (26.2)
Widowed	217	38 (17.5)
***Living with a partner^*a*^***		
Yes	2,794	994 (35.6)
No	655	23 (3.5)
***Work situation^*a*^***		
Employed	1,479	458 (31.0)
Unemployed	203	67 (33.0)
Retired	1,642	548 (33.4)
Other	215	54 (25.1)
**Medical factors**		
***Tumor location***		
Breast	840	210 (25.0)
Digestive organs	725	189 (26.1)
Male genital organs	630	363 (57.6)
Prostate	592	352 (59.5)
Testicles	34	11 (32.4)
Penis	4	0 (0.0)
Respiratory organs	303	84 (27.7)
Female genital organs	294	85 (28.9)
Ovar	115	31 (27.0)
Uterus	66	39 (59.1)
Vulva	29	10 (34.5)
Vagina	6	4 (66.7)
Hematological cancers	277	69 (24.9)
Urinary tract	199	61 (30.7)
Lip, oral cavity and pharynx	101	34 (33.7)
Skin	79	22 (27.9)
CNS (eye, brain, other parts)	66	19 (28.8)
Mesothelial and soft tissue	57	13 (22.8)
other	106	26 (24.5)
***Cancer care setting***		
Inpatient care	1,536	453 (29.5)
Outpatient care	1,228	413 (33.6)
Inpatient rehabilitation	913	304 (33.3)
***UICC tumor stage***		
I	547	154 (28.2)
II	643	236 (36.7)
III	505	170 (33.7)
IV	787	252 (32.0)
Uncertain^*b*^	1,142	337 (29.5)

*CNS, central nervous system; n, sub-sample size; UICC, Union for International Cancer Control; classifies the tumor development in five stages.^a^Based on a different sample size.^b^Classification between stage III and IV was not clearly determined at the time of assessment.*

### Frequencies of Sexual Problems Depending on Sociodemographic and Medical Factors

Out of 3,677 cancer patients, 1,170 (31.8%) reported sexual problems with almost twice as men (40.5%) as women (23.7%).

### Associations of Sexual Problems With Psychosocial Factors

Sexual problems were associated with higher levels of depression, anxiety and distress. Sexual problems were adversely associated with social support: In detail, positive support was negatively associated with sexual problems, whereas detrimental interactions were positively associated with sexual problems. Sexual problems were associated with lower quality of life in terms of physical, roles, cognitive, social, emotional functioning, and global health (Table 2).

**TABLE 2:** Means, standard deviations, and effect sizes.

	Sexual problems				
	“Yes”		“No”		
Variable	*n*	*M* (*SD*)	*n*	*M* (*SD*)	η^2^
	1,104		2,333		
Symptoms of depression		7.47 (4.94)		5.81 (4.33)	0.028
Symptoms of anxiety		6.49 (4.36)		4.52 (3.71)	0.052
Symptoms of distress		5.15 (2.55)		4.26 (2.53)	0.026
Positive support		13.37 (2.90)		13.80 (2.70)	0.005
Detrimental interaction		4.06 (3.10)		3.48 (3.11)	0.008
Physical functioning		67.52 (23.68)		72.43 (23.18)	0.010
Roles functioning		49.46 (34.43)		59.81 (34.56)	0.019
Cognitive functioning		68.30 (28.65)		79.30 (24.91)	0.037
Social functioning		50.51 (33.27)		66.19 (31.84)	0.049
Emotional functioning		56.04 (26.80)		69.03 (24.54)	0.054
Global health		50.82 (22.62)		58.96 (22.52)	0.028

*n, subsample size; M, mean; SD, standard deviation; η^2^, partial eta squared (0.01 = small effect, 0.059 = moderate effect, 0.138 = strong effect, Cohen, 1988); all tests were statistical significant with p < 0.001.*

### Independent Correlates of Sexual Problems

With respect to our final regression model, we found that sexual problems were significantly associated with male gender OR = 2.35, 95% CI [1.80–3.07] living with a partner OR = 2.83, 95% CI [2.17–3.70]. Sexual problems were significantly associated with elevated symptoms of anxiety OR = 1.05, 95% CI [1.02–1.10] and with lower levels of positive support OR = 0.93, 95% CI [0.90–0.97]. Our model showed significantly associations between sexual problems and lower emotional functioning, social functioning and cognitive functioning, OR = 0.99, 95% CI [0.99–1.00]. Sexual problems were significantly associated with some types of cancer (e.g., higher chance for cancer affecting male genital organs, OR = 2.65, 95% CI [1.78–3.95] and lower chance for cancer affecting digestive organs, OR = 0.60, 95% CI [0.42–0.85], whereas distress, symptoms of depression and type of therapy had no significant associations with sexual problems (Table 3).

**TABLE 3:** Multivariate hierarchical logistic regression of sexual problems with sociodemographic and psychosocial factors as determinants.

	Model 1	(*R*^2^ = 0.06)	Model 2	(*R*^2^ = 0.16)	Model 3	(*R*^2^ = 0.17)	Model 4	(*R*^2^ = 0.19)	Model 5	(*R*^2^ = 0.26)	Model 6	(*R*^2^ = 0.26)

	OR	95% CI	OR	95% CI	OR	95% CI	OR	95% CI	OR	95% CI	OR	95% CI
**Sociodemographic**												
Sex^a^	2.03*	1.73–2.38	2.61*	2.19–3.10	2.61*	2.20–3.11	2.71*	2.27–3.25	2.37*	1.82–3.10	2.35*	1.80–3.07
Age^b^	0.96	0.89–1.03	1.03	0.95–1.11	1.03	0.95–1.11	1.06	0.98–1.15	0.99	0.91–1.10	0.99	0.91–1.08
Living with a partner^c^	2.16*	1.72–2.70	2.54*	2.00–3.22	3.04*	2.35–3.92	2.82*	2.18–3.65	2.82*	2.16–3.67	2.83*	2.17–3.70
**Psychosocial**												
Distress^d^			1.05*	1.01–1.09	1.05*	1.01–1.09	1.02	0.98–1.06	1.02	0.98–1.07	1.02	0.98–1.07
Depressive symptoms^e^			1.04*	1.02–1.07	1.04*	1.02–1.06	1.01	0.98–1.03	1.02	0.99–1.05	1.02	0.99–1.05
Symptoms of anxiety^f^			1.11*	1.08–1.14	1.10*	1.08–1.13	1.06*	1.02–1.09	1.05*	1.02–1.09	1.05*	1.02–1.10
**Social support**												
Positive support^g^					0.93*	0.90–0.96	0.94*	0.91–0.97	0.93*	0.90–0.97	0.93*	0.90–0.97
Detrimental interaction^h^					1.01	0.98–1.04	1.00	0.97–1.03	1.01	0.98–1.04	1.01	0.98–1.04
**Quality of life**												
Global health^i^							1.00	0.99–1.01	1.00	0.99–1.00	1.00	0.99–1.00
Physical functioning^j^							1.00	0.98–1.01	1.00	0.99–1.01	1.00	1.00–1.01
Emotional functioning^k^							0.99*	0.98–0.99	0.99*	0.99–1.00	0.99*	0.99–1.00
Social functioning^l^							0.99*	0.98–0.99	0.99*	0.99–1.00	0.99*	0.99–1.00
Roles functioning^m^							0.99	0.99–1.99	1.00	0.99–1.00	1.00	1.00–1.01
Cognitive functioning^o^							0.99*	0.98–0.99	0.99*	0.99–1.00	0.99*	0.99–1.00
**Tumor location**												
Digestive organs									0.60*	0.44–0.83	0.60*	0.42–0.85
Male genital organs									2.46*	1.71–3.55	2.65*	1.78–3.95
Respiratory organs									0.61*	0.41–0.91	0.57*	0.37–0.87
Female genital organs									1.16	0.82–1.64	1.15	0.80–1.66
Blood and blood-forming organs									0.46*	0.30–0.68	0.43*	0.27–0.68
Urinary tract									0.74	0.48–1.16	0.81	0.50–1.30
Lip, oral cavity and pharynx									0.84	0.48–1.44	0.76	0.43–1.33
Skin									0.57	0.30–1.07	0.59	0.31–1.14
Eye. Brain, other parts of CNS									0.78	0.41–1.49	0.73	0.37–1.41
Mesothelial and soft tissues									0.36*	0.17–0.79	0.36*	0.16–0.79
**Type of therapy**												
Operation											0.91	0.72–1.15
Radiation therapy											1.17	0.97–1.41
Chemotherapy											1.10	0.90–1.35
Hormontherapy											0.87	0.66–1.15

CNS, central nervous system; 95% CI, 95% confidence interval of odds ratio; OR, odds ratio; R^2^, Nagelkerke R^2^. *Significant on a level of 0.05. ^a^(Female = 0, male = 1). ^b^Grouped. ^c^No = 0, yes = 1. ^d^Per 10 points. ^e–o^Per point.

## Discussion

In our cross-sectional multicenter study among a large and representative sample of patients across different tumor locations, around one-third reported having sexual problems. We found men reporting more sexual problems compared to women. Living with a partner and having a cancer diagnosis affecting the sexual organs were associated with sexual problems. As our results show, there was no significant differences between different age groups in the occurrence of sexual problems. We found that sexual problems were associated with significant higher levels of anxiety, depression and distress, with non-significance of the latter two factors when considered independently. Our results showed that sexual problems and positive support were negatively associated. Detrimental interactions were positively associated, with a non-significant association of the latter one, when considered independently in the final regression model. Sexual problems were also associated with lower quality of life in terms of physical, roles, cognitive, social, emotional functioning, and global health. In our regression model, merely the associations between sexual problems and positive support, cognitive function as well as emotional and social function remained significant.

Our findings are in line with a population-based study comparing *N* = 6,129 non-cancer controls with *N* = 651 cancer patients aged 50 and older. 29.8% of patients (most prevalent breast cancer, prostate cancer, colorectal cancer and melanoma) reported having concerns about sexual health in comparison to the cancer-free control group (Jackson et al., 2016). We found a similar rate of sexual problems in patients, within an even wider range of age (from 18 to 75 years). Our findings showed that sexual problems occur across all age groups, indicating the relevance of sexual health and corresponding issues in all stages of life. As previous findings show, sexual problems do both occur in older and younger cancer patients as they range from erectile dysfunction (Ellis et al., 2010; Sendur et al., 2014; Fekih-Romdhane et al., 2019) and impotence (Bailey et al., 2015) in older men, and lubrication disorders in older women (Milbury et al., 2013), as well as in younger cancer patients with both reproductive and non-reproductive cancer experiencing, sexual dissatisfaction and changes in sexuality (Mütsch et al., 2019).

Our results highlight the relevance of sexual problems for mental health. We found that symptoms of anxiety were significantly associated with sexual problems, distress and symptoms of depression were elevated but non-significant in the final regression model. Previous research has also shown that sexual problems due to cancer have negative effects on psychological well-being (Levin et al., 2010; Reese et al., 2018).

In our sample, cancer patients with sexual problems also significantly scored lower in both social and emotional functioning as indicators of quality of life in those areas. When enduring an illness like cancer, patients are more dependent on their loved ones and health professionals in the realm of receiving care and social and emotional support (Masterson et al., 2015). Social support is considered one of the most important resources for coping with stressful events, such as cancer. However, as recent studies show, interactions can also be unsupportive, namely detrimental to the patients’ well-being and dignity (Philipp et al., 2016). Our results show that cancer patients with sexual problems had significantly less positive social support and more detrimental social interactions. According to findings from one cross-sectional retrospective study with *N* = 710 cancer patients, those who were less able to cope with the situation emotionally had greater needs for psychosocial support (Ernstmann et al., 2009).

According to our findings, living with a partner was one of the most relevant factors for indicating sexual problems. We cannot assume that patients without partners do not have sexual problems. However, it could be that this group of cancer patients did not report the presence of sexual problems, because they register them less frequently or intensively. However, we can state that cancer patients who live in a partnership are also more frequently confronted with sexual problems that can occur in the context of cancer treatment and thus influencing partnership and intimacy.

Previous findings showed that patients with cancer often tend to neglect sexual life due to changes in their physical appearances, integrity, and function of the body (Manier et al., 2018). Within the scope of our results, those tumor locations, directly affecting the sexual organs had a higher chance for sexual problems, whereas others like colorectal cancers had a lower chance for sexual problems. This might seem obvious, since changes in the reproductive structures, whether they are due to hormonal or physiological changes, could impede sexual activity (Rhoten, 2016).

### Clinical Implications

Our findings emphasizes the notion that sexuality should be viewed as a health issue that has an impact on quality of life and other factors detrimental or beneficial to the overall health and well-being of cancer patients. Accordingly, it seems that management of psychological and mental well-being in cancer patients is crucial when it comes to treatment and recovery process (Twitchell et al., 2019).

As our data might show, it is therefore wrong to assume that cancer patients have no concerns about their sexual health. The occurrence of sexual problems in cancer patients indicates the importance of those being able to talk about their concerns, as previous studies show that often those concerns and the conversations about them are considered as a taboo in cancer care (Redelman, 2008; Nyatanga, 2012). Within a study with lymphoma patients data from *N* = 466 respondents were gathered, where only 16% of patients stated having discussed sexuality with their oncologists or caregivers (Arden-Close et al., 2011). This is contrary to patients’ needs in discussing such issues. According to another study within the scope of sexual health needs for women with cancer, 70% of the cohort were concerned about sexual function and preferred the topic to be raised by the medical team (Stabile et al., 2017).

A lack of communication on that specific topic often results from a lack of background knowledge and comfort levels to engage in discussions about sexual issues (Boswell and Dizon, 2015). These shortcomings arise on both sides, when willingness to discuss such intimate topics is little. Communication on sexual health during cancer care therefore is vital and should be included in the routine assessment of other physical and psychological symptoms. A screening tool applied on a regular basis holds the potential to identify cancer patients with elevated risk on behalf of sociodemographic (like gender, relationship status), medical (like tumor location) and psychosocial factors (like symptoms of anxiety) and of patients’ concerns. This might help to sensitize patients and health professionals to build up communication on sexual health. Further, psychoeducative interventions, where cancer patients get access to high-quality information about the short and long-term effects of cancer and its treatment are helpful (Canty et al., 2019). In addition, careful consideration of treatment options, particularly in the context of surgical procedures that may be associated with impaired sexual physiology, is essential (Strauß, 2016). Healthcare professionals should guide and prepare their patients to have sufficient information about treatments and possible side effects, including sexual health disruptions that may persist even into survivorship (Canty et al., 2019).

### Limitations

Although our study is based on a large and representative sample of cancer patients, we measured sexual problems only via one item, thus a detailed investigation of the type of the sexual problems needs to be determined in future studies. However, identifying sexual problems via one-single item in a routine screening could act as an entry for further examination on sexual health and related problems. The practitioner might use this brief information to check for more symptoms of possible impairment on patients’ sexuality.

Secondly, the cross-sectional study design did not allow interferences on causality, thus further studies on longitudinal effects should be conducted.

In our study, frequency of occurrence estimation was based on self-reports. Sexual health and its vulnerability is an issue prone to stigmatization. It is also possible that self-reported data is biased toward underestimation or is a subject to social acceptability bias. For this topic, however, it is to note that this issue may be disguised by patients in personal interviews and thus the assessment via self-report may provide even more valid data.

Lastly, our sample was slightly biased toward younger age, more education and rehabilitation setting, therefore limited in generalizability.

## Conclusion

In this study among cancer patients, a large proportion reported to have sexual problems. Male patients and those living with a partner had elevated levels for such problems. Sexual problems were associated with various variables on well-being. Longitudinal studies are needed to confirm our findings regarding the relevance and risk groups of this issue in cancer patients and survivors.

## Data Availability Statement

The data analyzed in this study is subject to the following licenses/restrictions: The data that support the findings of this study are available on request from the corresponding author. The data are not publicly available due to privacy or ethical restrictions. Requests to access these datasets should be directed to corresponding author.

## Ethics Statement

The studies involving human participants were reviewed and approved by the Ethics Committees of all participating centers (file numbers: Freiburg 244/07; Hamburg: 2768; Heidelberg: S-228/2007-50 155 039; Leipzig: 200-2007; Schleswig-Holstein: 61/09; and Würzburg: 107/07). The patients/participants provided their written informed consent to participate in this study.

## Author Contributions

AM-T and SH had the initial idea for this study. AM-T collected the data based upon the study. SH wrote the first draft of the manuscript. SH and MF performed statistical analyses. PE and KG did major revision on all sections of the manuscript. All authors contributed to manuscript revision, read, and approved the submitted version.

## Conflict of Interest

The authors declare that the research was conducted in the absence of any commercial or financial relationships that could be construed as a potential conflict of interest.
